# Network Centrality Analysis in Fungi Reveals Complex Regulation of Lost and Gained Genes

**DOI:** 10.1371/journal.pone.0169459

**Published:** 2017-01-03

**Authors:** Jasmin Coulombe-Huntington, Yu Xia

**Affiliations:** 1 Institute for Research in Immunology and Cancer, University of Montreal, Montreal, Quebec, Canada; 2 Department of Bioengineering, Faculty of Engineering, McGill University, Montreal, Quebec, Canada; Michigan State University, UNITED STATES

## Abstract

Gene gain and loss shape both proteomes and the networks they form. The increasing availability of closely related sequenced genomes and of genome-wide network data should enable a better understanding of the evolutionary forces driving gene gain, gene loss and evolutionary network rewiring. Using orthology mappings across 23 ascomycete fungi genomes, we identified proteins that were lost, gained or universally conserved across the tree, enabling us to compare genes across all stages of their life-cycle. Based on a collection of genome-wide network and gene expression datasets from baker’s yeast, as well as a few from fission yeast, we found that gene loss is more strongly associated with network and expression features of closely related species than that of distant species, consistent with the evolutionary modulation of gene loss propensity through network rewiring. We also discovered that lost and gained genes, as compared to universally conserved “core” genes, have more regulators, more complex expression patterns and are much more likely to encode for transcription factors. Finally, we found that the relative rate of network integration of new genes into the different types of networks agrees with experimentally measured rates of network rewiring. This systems-level view of the life-cycle of eukaryotic genes suggests that the gain and loss of genes is tightly coupled to the gain and loss of network interactions, that lineage-specific adaptations drive regulatory complexity and that the relative rates of integration of new genes are consistent with network rewiring rates.

## Introduction

Gene gain and loss are very important components of evolution and interspecies differences. For example, a dozen distant eukaryotes have been shown to share as little as 9% of their combined gene families [1]. Proteomes are constantly evolving and the dynamics of gene gain and loss processes shape the networks of interactions that determine the behavior of higher-level systems. Unlike protein sequence evolution, which provides an informative evolutionary landscape over the length of a single protein, the study of gene gain and loss necessitates a genomic-level view and many species.

The set of genes which are universally conserved across a phylogenetic tree has been termed the “core” genome of the lineage [2, 3]. Studies of gene loss comparing distant eukaryotes have shown that lost genes differ significantly from core genes in many ways. Lost genes, in species where they are present, have fewer protein-protein interaction (PPI) partners, lower mRNA expression, lower sequence conservation and their deletion is less likely to produce a lethal phenotype, known as gene essentiality [4]. Studies on horizontally transferred genes, *de novo* gene birth, and gene duplication have shown similar features for gained genes, with the most recently gained genes harboring the most extreme values [5–7]. Gene copy number volatility has also been shown to correlate negatively with genetic interaction degree [8], but no distinction was made between gene loss, gene gain and gene duplication events.

The transcriptional regulatory network is known to rewire faster than other biological networks [9] and it has been shown that recently transferred genes in prokaryotes acquire new regulators much more quickly than they do PPI partners [5]. Apart from this rapid initial gain of regulators in the first ~20–40 million years, the longer-term trends in the regulatory network rewiring that follows gene gain have not been studied in depth. A study on *de novo* gene birth in yeast suggested that older genes were more likely to possess at least one regulator, but the network used in the analysis was restricted to a single high-throughput study, and to canonical transcription factor binding sites (TFBSs) conserved across *sensu stricto* Saccharomyces species, systematically excluding most TFBSs in younger promoters. The relationship between gene loss and regulatory network structure has to our knowledge never been studied, except for a recent paper of ours identifying a correlation between the evolutionary rate of transcription factors and the lineage-specificity of their target genes [10].

Studies into gene gain have established the time-dependence of gene integration processes [5, 7, 6]. Gene loss, however, has not yet been analyzed from a temporal perspective. Gene loss propensity has typically been viewed as an inherent property of the genes themselves, and was therefore modeled as a constant value, averaged over the entire phylogenetic tree [4, 8]. It is now well established that the relative importance of genes is influenced by their position in the different biological networks [11, 10, 8] and given that networks evolve over time, we may expect gene loss events to be preceded by a phase of network marginalization. Here, we investigate whether gene loss propensity could be modelled more accurately as a branch-specific property, consistent with the influence of evolutionary network rewiring.

Gene duplication, including whole-genome duplication, is one of the most common mechanisms for gene gain in eukaryotes [12, 13]. However, there is an important functional distinction between gene duplication, which merely increases the number of genes in a family, and horizontal gene transfer or *de novo* gene birth, which can introduce an entirely new gene family into a genome. Duplication produces new copies of genes which are in many ways already integrated into the networks and functional organization of the cell and at least one of the copies must likely uphold the functions of the parent gene. For these reasons, we considered duplication events separately from other gene gain events and distinguished between the slowest evolving copy of a set of duplicated genes and the other copies, which are expected to be relatively free of the functional constraints of the parent gene [14–17, 12]. Furthermore, since duplicated genes have already been studied in much more depth than other gained genes, including in a network context [14, 15, 18, 16, 17, 19–21], this study does not cover all of their network features.

## Results

### Identifying gene loss and gain

**Fig 1:**
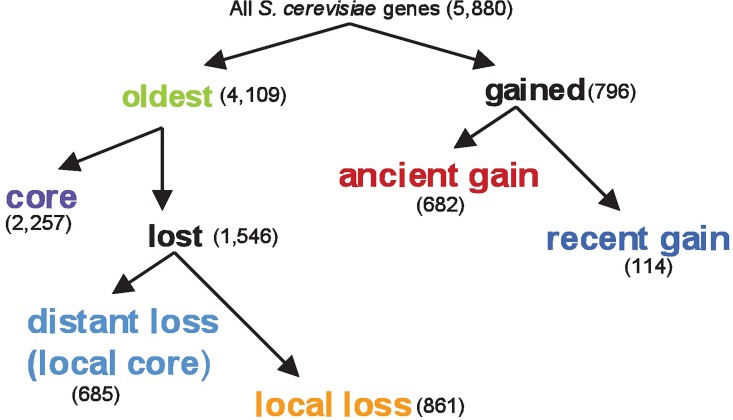
Flowchart depicting how genes were classified into different life stages. Numbers in parentheses indicate the number of genes in each category. For reasons explained in the first paragraph of the results section, not all *S*. *cerevisiae* genes could be classified in either the “gained” or “oldest” categories. While gene loss also affects younger genes, we restricted the analysis of gene loss to the oldest genes, in order to control for the effects of gene age.

**Fig 2:**
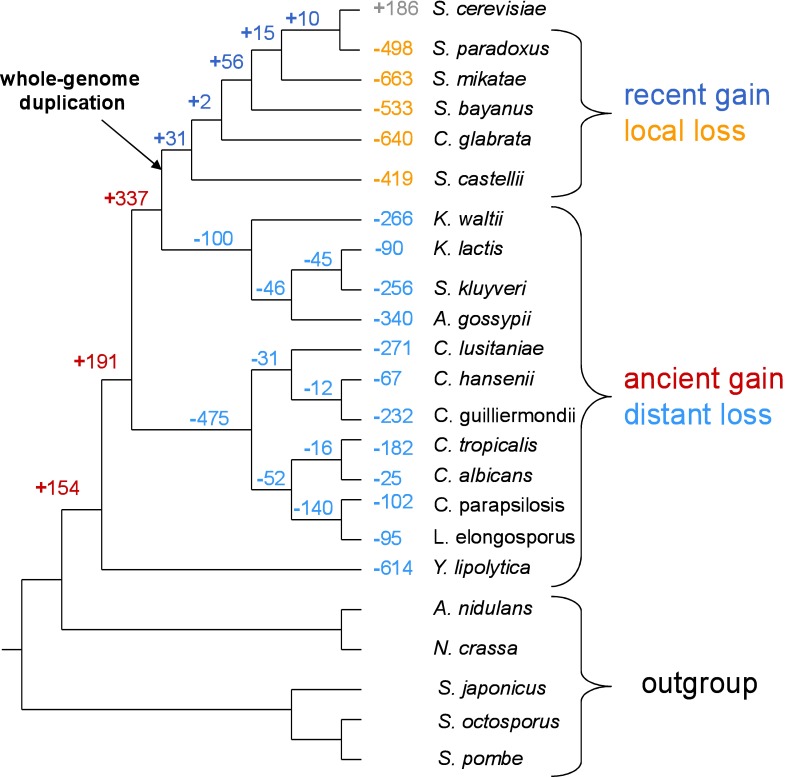
Inferred gene loss and gain events displayed along the yeast phylogenetic tree and how we inferred the life stage of different genes based on the phylogenetic location of their loss or gain events. The “+” sign denotes gains and the “-”sign, losses. Recent gains and local losses were defined as those having occurred after the split with *K*. *waltii*, with the exception of genes gained in *S*. *cerevisiae*.

Using gene orthology assignments across 23 ascomycete fungi genomes from the Orthogroups database [22], we classified all *S*. *cerevisiae* protein-coding genes according to their representation across the tree (Figs 1 and 2). Only genes with at least one ortholog in a second species were considered, in order to avoid including false positive ORF predictions, not actually coding for functional proteins. Genes which possess one or more orthologs in the outgroup species (see Methods) were considered to be the oldest genes. The remaining genes, those which possess no ortholog in any of the outgroup species, were identified as likely having been gained along the *S*. *cerevisiae* lineage and were further sub-classified according to their estimated age. This group includes 796 genes, after we filtered out potential gene duplication events (see Methods). The oldest genes were classified as either being universally conserved or lost in one or more species, except for those lost in one or more outgroup species. Many of the genes gained since the divergence from *N*. *crassa* were also found to be lost in one or more species, but only loss events affecting the oldest genes were considered in order to avoid confounding the properties associated with gene loss from those associated with gene gain. 2,257 of the oldest genes possessed one or more orthologs in all 23 species of the tree and were thus considered the universally conserved, or “core”, genome. Starting from the roughly 350 million year old divergence of the *N*. *crassa* lineage [23], we identified 3,718 gene loss events, implicating orthologs of 1,546 of the oldest genes in *S*. *cerevisiae* (see Methods for details). Lost genes were then sub-classified according to the phylogenetic distance from *S*. *cerevisiae* of the closest loss event. Fig 2 shows the number of loss and gain events on each branch of the tree.

### The effects of whole-genome duplication on gene gain and loss rates

Whole-genome duplication (WGD) events lead to the creation of a large number of new genes, many of which are lost shortly thereafter [24–27] while many others assume novel functions [28, 27]. As we expect based on these earlier findings, we observe a 4 fold increase in the rate of gene loss along the *S*. *cerevisiae* lineage following the whole-genome duplication (WGD) event (Fig 2), considering relative branch lengths (see Methods). Interestingly we also observed a 5.3 fold reduction in the rate of gene gain (Fisher’s exact test *p* = 2.2x10^-86^). This observation is likely the result of new genes created during the WGD assuming new functions which would otherwise have been fulfilled by other genes, including new genes gained by other mechanisms.

### Number of regulators of lost and gained genes

**Fig 3:**
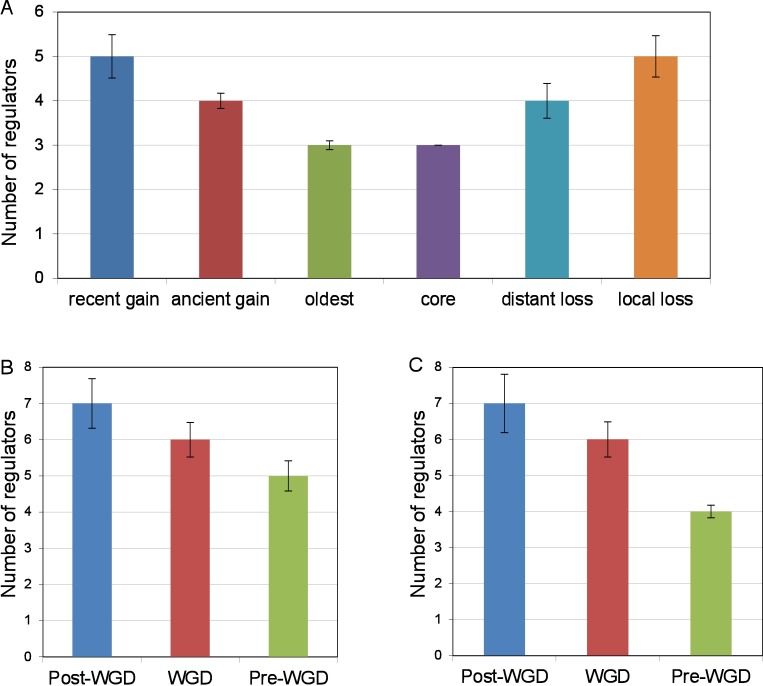
Median regulatory in-degree, based on all studies compiled by the YEASTRCACT database [29], for (A) each of the evolutionary life-stages of genes, (B) genes duplicated before (Pre-WGD), during (WGD), or after (Post-WGD) the whole-genome duplication event, excluding the slowest evolving copy, and (C) the slowest evolving copy of each duplicated gene set. Error bars show the bootstrapped standard error of the median based on 100 resamplings.

While it has been shown that lost and gained genes possess fewer protein-protein interactions, genetic interactions and higher average expression than universally conserved core genes [5, 6, 4], the relationship between transcriptional regulatory network structure and gene gain and loss has not been studied as extensively. The regulatory network is known to rewire more rapidly than most other biological networks [9] and may thus play a relatively more active role in the integration of new genes as well as the regulation of lineage-specific genes. Based on a collection of high-throughput and small-scale studies [29], we found to our surprise that universally conserved genes have significantly fewer transcriptional regulators (regulatory in-degree) than lost genes (Wilcoxon rank sum test *p* = 9.3x10^-22^; Fig 3A) and the oldest genes similarly have fewer regulators than gained genes (*p* = 6.6x10^-5^; Fig 3A). We found that duplicated genes show a similar trend. Comparing genes duplicated before the whole-genome duplication (pre-WGD) to genes duplicated after (post-WGD), we found that the number of regulators tends to decrease over time following the duplication event, affecting both the faster evolving copies (Wilcoxon test *p* = 0.0015, Fig 3B) as well as the slowest evolving copy of each set of duplicate genes (Wilcoxon test *p* = 9.2x10^-7^, Fig 3C, see Methods). This suggests that the subfunctionalization or neofunctionalizatoin of young duplicate genes is accompanied by increased regulatory complexity, similarly to the integration phase of genes gained by other mechanisms. The relative centrality of lost and gained genes in the regulatory network contrasts sharply with the trend observed for genetic interaction and PPI networks [4, 8]. It indicates a strong plasticity of transcriptional networks and that complex regulation may be an inherent property of lineage-specific gene regulation.

It is possible that the greater number of TFBSs occurring in the promoters of gained and lost genes could be the result of reduced selective pressure allowing spurious TFBSs to arise by chance. To address this possibility, we considered the number of TFBSs in the promoters of the newest genes, present only in *S*. *cerevisiae*. These promoters have had the least time to evolve and should thus possess a TFBS density most representative of a complete absence of selective constraint. If older gained genes or lost genes have numbers of regulators which significantly exceed this number, this would suggest that at least a fraction of these TFBSs must be maintained by selective pressures. We limited the analysis to genes with one of more regulators in the network in order to ensure that they were included in regulatory network mapping studies. We found that genes gained only in *S*. *cerevisiae* have a lower number of transcriptional regulators on average than recently gained genes shared by at least one other species (Wilcoxon test p = 2.1x10^-8^) or than locally lost genes (Wilcoxon test p = 1.1x10^-7^). This rapid initial gain of regulators, which has also been observed in another study [5], suggests that the high regulatory in-degree of lineage-specific genes is a feature which is actively selected for.

### Condition specificity of lost and gained genes

**Fig 4:**
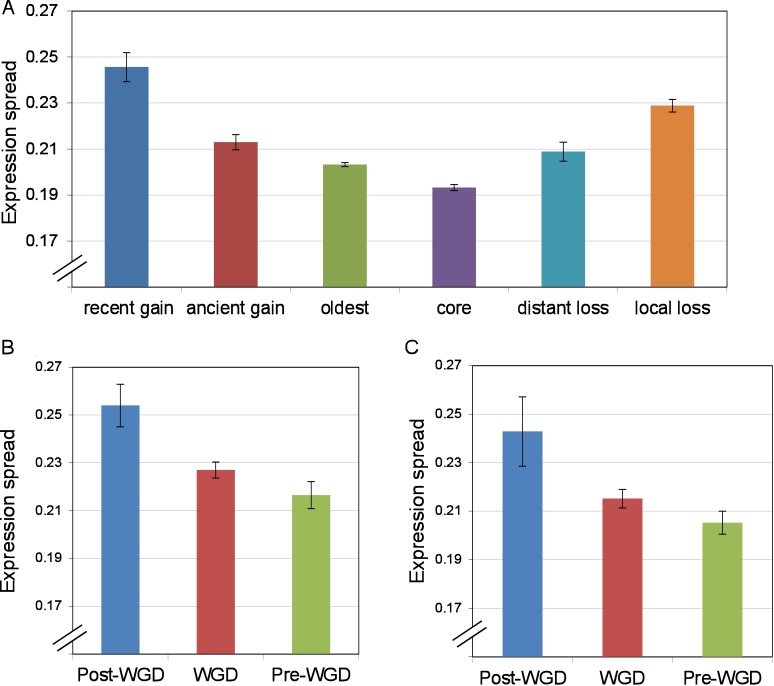
Median expression spread, the standard deviation of log_10_ microarray probe fluorescence intensities across 300 conditions and chemical treatments [33], shown for (A) each of the evolutionary life-stages of genes, (B) duplicated genes, excluding the slowest-evolving copy and (C) the slowest-evolving copy of duplicated gene sets. Error bars show the bootstrapped standard error of the median based on 100 resamplings.

High regulatory in-degree (possessing many regulators) and highly conserved promoter regions have been associated with higher expression variability [30–32]. Lost or gained genes, being found only in a subset of species, are likely to encode for conditionally expressed functions, requiring relatively complex expression level regulation. Stress-related genes, for example, have been shown to be enriched in lost and duplicated genes [20]. Furthermore, the complex transcriptional regulatory program of newly gained genes may allow the cell to tightly regulate their abundance and time of expression, minimizing energetic costs and potentially unfavorable interactions, as they more slowly become integrated into the other types of networks. In order to estimate the expression variability of genes, we retrieved yeast expression data measured under 300 different conditions and chemical treatments [33] and calculated the standard deviation of expression levels for each gene. We found that lost and gained genes have significantly more variable expression levels across conditions than core genes (Wilcoxon test *p*<5.1x10^-36^; Fig 4A) and recently gained genes possess more variable expression levels than the oldest genes (Wilcoxon test *p* = 1.2x10^-6^; Fig 4A). These relationships remain significant when controlling for differences in average expression level using multivariate linear regression (partial F-test; lost genes *p*<2x10^-16^; gained genes *p* = 1.1x10^-4^). Duplicated genes have already been shown in earlier works to be more conditionally expressed than their non-duplicated counterparts [14, 34] and our results confirm that this is the case, affecting both fast (Wilcoxon test *p*<2.1x10^-5^, Fig 4B) and slow evolving copies (Wilcoxon test *p* = 1.6x10^-3^, Fig 4B). These results suggest that both lost genes and recently gained genes tend to be expressed in a condition-specific manner, potentially explaining why they possess more transcriptional regulators.

### Gain and loss of transcription factors

Given the highly active role of the transcriptional network in the regulation of lost and gained genes, we decided to explore the role of gene gain and loss in *trans-*regulatory network evolution. Using the list of *S*. *cerevisiae* transcription factors (TFs) compiled in Wang et al. [35], we found that TFs are highly enriched in all types of lineage-specific genes (Table 1). Specifically, we found that lost genes among the oldest genes contain proportionally 2.2 fold more TFs than core genes (Fisher’s exact test *p* = 1.4x10^-3^), gained genes 2.3 fold more than the oldest genes (Fisher’s exact test *p* = 9.0x10^-5^) and duplicated genes 2.8 fold more than non-duplicated genes (Fisher’s exact test *p* = 3.6x10^-9^). These results suggest that *trans-*regulatory network evolution plays a central role in lineage-specific adaptation.

**Table 1:** Transcription factor enrichment in lost and gained genes.

	Core	Lost	Gained	Duplicated
Number of TFs	26	40	35	80
Percent TFs	1.2	2.6	4.4	4.5
P-value*	-	1.4x10^-3^	9.0x10^-5^	3.6x10^-9^

*:based on Fisher’s exact test.

### Network marginalization as a lineage-specific predictor of gene loss

**Fig 5:**
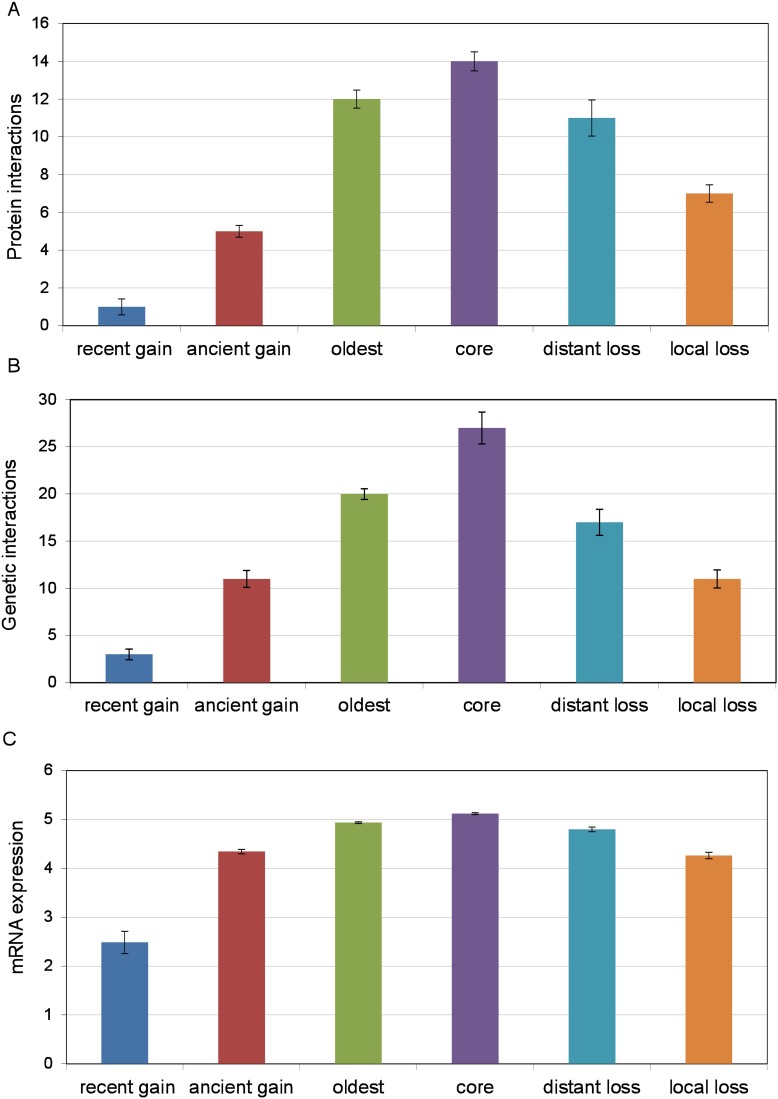
Known properties of lost and gained genes, shown for each of the evolutionary life stages of genes, including (A) median PPI degree, as compiled by the *Saccharomyces* Genome Database [40], (B) median genetic interaction degree, as compiled by the *Saccharomyces* Genome Database [40], excluding essential genes [49], and (C) median mRNA expression level, as represented by the log_e_ of RNA-seq read counts in rich media [41]. Error bars show the bootstrapped standard error of the median based on 100 resamplings.

In earlier studies, the propensity for gene loss was modeled as an unchanging inherent property of a gene [4, 8]. Underlying this model is the implicit assumption that network structure is either static throughout evolution, or that network rewiring has no influence on a gene's propensity to be lost. Here, we investigate the possibility that gene loss propensity could be modeled more accurately as a branch-specific property. Within the set of oldest genes lost in one or more species, we distinguished between genes lost only in distant species (distant loss, Figs 1 and 2), considered the "local core" genome, from the locally volatile genes, lost in closely related species (local loss, Figs 1 and 2). The two categories of lost genes are of the same age group and have comparable propensity for gene loss when averaged over the entire tree (Wilcoxon test p = 0.90, see Methods), differing only by the phylogenetic distance of the closest species where the gene was lost. As shown in Figs 5, 3A and 4A, genes lost in close species have stronger network and expressional signatures of marginalization than genes lost only in distant species. Specifically, we found that ancestral genes lost in species close to *S*. *cerevisiae* have significantly lower PPI interaction degree (Wilcoxon test p = 8.4x10^-7^; Fig 5A), lower genetic interaction degree (Wilcoxon test p = 6.7x10^-6^; Fig 5B), lower mRNA expression (Wilcoxon test p = 2.3x10^-9^, Fig 5C), higher expression variability (Wilcoxon test p = 2.9x10^-5^, Fig 4A) and higher regulatory in-degree (Wilcoxon test p = 4.3x10^-4^, Fig 3A), than genes lost solely on distant branches. Gene essentiality shows a similar trend but the difference is only marginally significant (Fisher's exact test, p = 0.047, data not shown). These significant differences show that the gene loss process in species close to *S*. *cerevisiae* is more closely tied to the network structure and expression levels found in *S*. *cerevisiae* than gene loss in distant lineages, consistent with gene-loss propensity being influenced by lineage-specific network rewiring.

**Fig 6:**
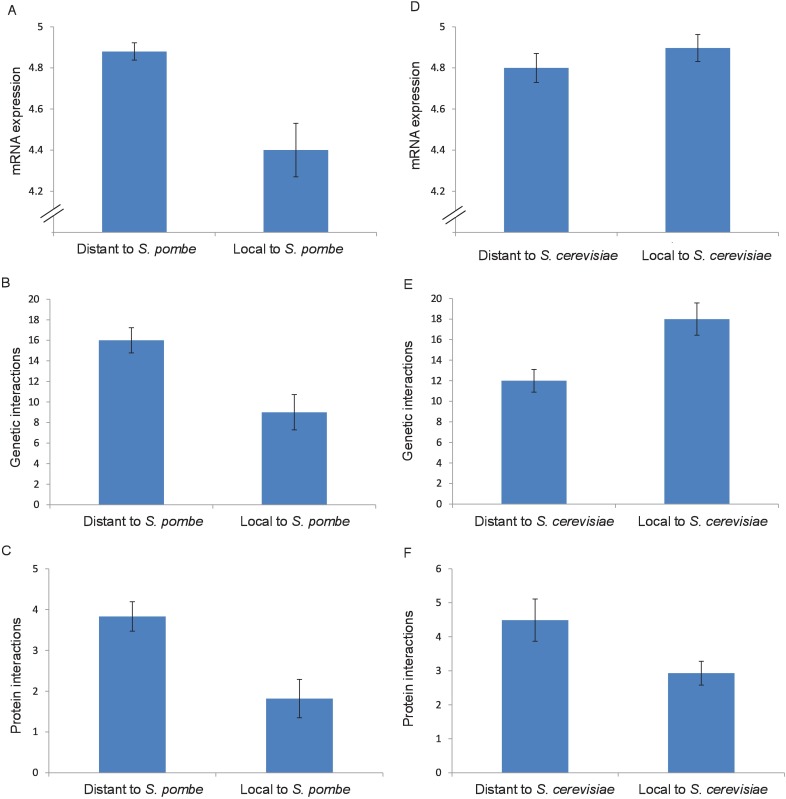
Properties in *S*. *pombe* of genes lost along different parts of the tree. A-C) Comparison of genes lost in species close to *S*. *pombe* (*S*. *octosporus* or *S*. *japonicus*) to genes lost along other branches, showing (A) median mRNA expression in *S*. *pombe*, (B) median genetic interaction degree in *S*. *pombe* and (C) mean PPI degree in *S*. *pombe*. D-F) Comparison of *S*. *pombe* orthologs of genes lost in species close to *S*. *cerevisiae* (after the divergence from *K*. *waltii*) to those of genes lost along other branches, showing (D) median mRNA expression in *S*. *pombe*, (E) median genetic interaction degree in *S*. *pombe* and (F) mean PPI degree in *S*.*pombe*. Error bars around medians show the bootstrapped standard error of the median based on 100 resamplings.

To confirm that these differences are not simply the result of biases in the gene loss process of the lineage, we acquired mRNA expression, genetic interaction data and protein-protein interaction data from *S*. *pombe* (see Methods), one of the most distant species from *S*. *cerevisiae* in the Orthogroups database. We found that genes lost in species close to *S*. *pombe*, i.e. *S*. *octosporus* or *S*. *japonicus*, possess significantly lower expression levels (Fig 6A, Wilcoxon test p = 4.1x10^-5^), genetic interaction degree (Fig 6B, Wilcoxon test p = 0.012) and protein interaction degree (Fig 6C, Wilcoxon test p = 1.7x10^-6^) in *S*. *pombe* than genes lost in other parts of the tree, demonstrating that the trends we observed for the *S*. *cerevisiae* lineage are not unique to the lineage. The fact that the genes lost in each lineage consistently correspond to those with relatively lower expression and fewer genetic and physical interactions is consistent with a scenario whereby gene loss propensity evolves over time and that increased gene volatility is accompanied by a process of functional marginalization through network rewiring. However, since the genes lost in each lineage are not the same sets of genes, we have not observed this marginalization process directly. To address this, we used the *S*. *pombe* data to compare the properties of the genes lost locally or distally to *S*. *cerevisiae*, taking care to include the same genes as those used for the comparisons with *S*. *cerevisiae* data. We found that genes lost close to *S*. *cerevisiae* show comparable expression levels (Fig 6D, Wilcoxon test p = 0.20), higher genetic interaction degree (Fig 6E, Wilcoxon test p = 0.012) and comparable protein interaction degree (Fig 6F, Wilcoxon test p = 0.076) in *S*. *pombe* than genes lost distantly to *S*. *cerevisiae*, contrasting with the trends observed using *S*. *cerevisiae* data (Fig 5B and 5C). The relatively low expression, genetic interaction degree and PPI degree of genes lost locally to *S*. *cerevisiae* are properties which thus appear to have been acquired specifically in the lineage, suggesting that a phase of network marginalization and decreasing expression tends to accompany gene loss in the *S*. *cerevisiae* lineage. On a side note, the relatively higher genetic interaction degree in *S*. *pombe* of genes lost locally to *S*. *cerevisiae* suggests that the increased rate of gene loss observed after the whole-genome duplication may have allowed for the loss of ancestrally more central genes as compared to gene loss in other lineages.

In order to better quantify the relative influence of the different factors considered on the propensity for gene loss in species close to *S*. *cerevisiae* and to identify potential co-dependencies between them, we applied multivariate logistic regression. Considering only the oldest genes, we classified each gene as having been lost or not in a species close to *S*. *cerevisae*, defined as after divergence from *K*. *waltii*. We then combined each of the following variables into a single logistic regression model: PPI degree, genetic interaction degree, the number of transcriptional regulators, mRNA expression level, expression variation and essentiality. The results, shown in Table 2, show that each one of these variables contributes significantly and independently to the prediction, except for PPI degree, which shows co-dependency with both genetic interaction degree and essentiality. This demonstrates that most of the associations observed are not merely artifacts of co-dependencies between features and confirms that regulatory in-degree and condition-specific expression are independent correlates of local gene loss propensity. The gene loss probabilities fitted by the model correlate with observed gene loss events with an R-squared of 0.12, indicating that 12% of the variability in the propensity for gene loss is effectively explained by this combination of network and expression features.

**Table 2:** Results of multi-variate logistic regression for predicting local gene loss.

Feature	PPI degree	Genetic degree	Regulatory in-degree	mRNA expression	Expression variation	Essentiality
Direction	-	-	+	-	+	-
p-value*	0.50	2.6x10^-11^	5.7x10^-8^	<2.2x10^-16^	5.0x10^-7^	2.0x10^-13^

*: p-value is based on the F-test, testing whether the model with the variable results in a significantly better fit than the nested model with all the other variables.

### Gene integration and evolutionary rewiring rates

It would be difficult to isolate the relative contribution of network rewiring and that of selective gene loss in the integration of new genes. However, we can ask whether there is an agreement between the relative rates of network integration and experimentally measured rates of network rewiring. What we mean specifically by the rate of network integration is the average rate at which new genes gain interactions in the network. Differences between the average network degree of genes from two different age groups should be explained by the rate of network integration and by potential biases in the loss of new genes. We compared the average network degree of anciently gained genes, which have had a limited time to gain interactions, to that of the oldest genes, which have had significantly more time to integrate, using the ratio of the two averages to represent the relative rate of network integration. We used the ratio of the two averages rather than the difference in order to normalize out the edge density, which can vary wildly across different types of networks. We used the anciently gained genes as the younger age group for this analysis because, as compared to the recently gained genes, these genes are more numerous and are less likely to be lost over time, which could bias the differences between age groups. The measured degree ratios order the different types of interactions, from fast to slow rate of gain, in the following order: transcriptional regulatory interactions, kinase interactions, genetic interactions, and PPIs, where kinase interaction degree was calculated as the number of PPI partners annotated with the GO term “protein kinases” [36]. This ordering follows exactly the order established by experimental measures of evolutionary network rewiring rates [9], which is unlikely the result of chance, given 24 possible orderings (*p* = 0.042). This observation is consistent with a model whereby new genes gain interactions over time through evolutionary network rewiring.

## Discussion

In this study, we have explored the role of network structure and rewiring in modulating the propensity for gene loss across phylogenetic lineages and shown that the rate of network integration of new genes tends to follow experimentally measured rates of network rewiring. We have also discovered that lost, gained, and duplicated genes, possess more complex transcriptional regulation and are more likely to be involved in transcriptional regulation than universally conserved genes. Consistent with this finding, we have also shown that these genes possess more complex expression profiles than core genes, providing a potential explanation for their more complex regulation.

Considering how lost and gained genes tend to possess much fewer PPI partners and genetic interaction partners than core genes, it may seem surprising that they tend to possess more regulators. However, previous works have established that the regulatory network possesses features suggesting an “inverted” structure relative to the PPI network and other cellular networks. For example, it was shown in yeast that TFs with more regulators tend to evolve faster than other TFs [37, 35], while this trend does not hold for generic genes [35, 10]. Furthermore, high regulatory in-degree and strong promoter conservation have been associated with condition-specific expression [31, 32] and with lower PPI network centrality [38]. These trends suggest that specialized, condition-specific functions generally require more complex regulation than do core housekeeping functions. This model is consistent with our novel observations that lost and gained genes possess more regulators and more complex expression programs than universally-shared core genes. Together with the finding that TFs show a strong tendency to be lost, gained or duplicated throughout evolution, our results suggests that lineage-specific adaptations may be the main driver of regulatory network complexity in yeast.

We have also found that the increased number of duplicate genes created by the whole-genome duplication following *S*. *cerevisiae*’s divergence from *K*. *waltii*, has had a significant effect on the subsequent rates of gene gain by other mechanisms, suggesting newly duplicated genes compete with other gained genes to fulfill a limited number of naturally selected functions. While the accelerated rate of loss of duplicated genes following whole-genome duplication has been well documented [24, 27, 28, 26], to our knowledge, no study had considered the impact of whole-genome duplication on the subsequent rates of gene gain.

These results teach us not only about the evolutionary processes surrounding gene gain and loss but also about the organization of biological networks themselves. Proteomes and networks are constantly evolving and are therefore best understood in an evolutionary context. Here, we have shown that the evolutionary dynamics of nodes and edges in biological networks are strongly inter-related. Every stage along the gene evolutionary life-cycle is associated with different network properties, shedding light on the etiology of important topological features of biological networks, such as their scale-free degree distribution [39].

## Methods

### Data collection

We downloaded the orthology mappings provided by the Orthogroups database [22]. PPI, genetic and regulatory interaction network data were retrieved from the *Saccharomyces* Genome Database [40]. Gene/proteins with no reported interactions were assigned an interaction degree of zero. mRNA expression information used to estimate “normal” expression was downloaded from the Gene Expression Omnibus database (http://www.ncbi.nlm.nih.gov/geo/) (Accession: GSE13750) and based on RNA-seq performed on yeast grown in rich media [41]. mRNA expression levels for 300 conditions [33], used to estimate condition-specificity, were downloaded from ExpressDB (http://arep.med.harvard.edu/ExpressDB/EDS45/). *S*. *pombe* mRNA expression information was downloaded from the Gene Expression Omnibus database (Accession: GSM74501) and absolute fluorescence scores representing logarithmically-growing wild-type cells were used [42]. Genetic interaction data for *S*. *pombe* was retrieved from the BioGRID database [43] and was restricted to the single largest high-throughput study [44] and only genes with at least one reported interaction were considered to ensure that each gene was included in the screen. PPI network data for *S*. *pombe* was retrieved from the BioGRID database. Genes with no reported interactions were assigned a degree of zero. Investigator bias in this case should be limited by the fact that all possible pairwise PPIs in *S*. *pombe* were recently screened via yeast-two-hybrid assay [45], in a study included in BioGRID.

### Identifying gene loss and gain events

We used the orthology mappings provided by the Orthogroups [22] database covering 23 fungal species, as well as the phylogenic tree from the same source. Aiming to study the features of lost and gained genes in *S*. *cerevisiae*, we only considered genes which are present in *S*. *cerevisiae*. We therefore only identified loss events which happened on branches leading away from *S*. *cerevisiae* and gain events on branches ancestral to *S*. *cerevisiae*. Gene gain and loss events identification was based on the Dollo parsimony model, i.e. minimizing the number of evolutionary events, assumed to be irreversible [46]. Species belonging to the two outer-most branches were used as the outgroup for the identification of gene loss and gain events, allowing newly gained genes to be distinguished from older genes with sparse representation (parallel loss events). In order to identify gene loss events, we identified proteins which were present in a common ancestor and missing in a descendant species. Assuming that a gene cannot be gained more than once independently, we defined gained genes as those found in the *S*. *cerevisiae* lineage but missing an ortholog in all outgroup species. We excluded genes specific to *S*. *cerevisiae*, which may not all encode for genuine functions. For each gene, the most distant species from *S*. *cerevisiae* to possess an ortholog was used to determine the age-group of the gain event.

### Identifying duplicated genes from the orthology map

Duplicated genes were defined as those for which an ortholog in another species maps to two or more genes in *S*. *cerevisiae*. For each pair or family of duplicates, we identify the slowest-evolving copy as the paralog with the highest level of sequence similarity to the ortholog in the closest species not affected by the duplication event. Other copies were considered the fast-evolving copies of the parent gene.

### Identifying potential duplications missed in orthology map

Gene duplication events do not lead to an increase in the number of gene families and were therefore discarded from the set of gene gains. While the Orthogroups data structure clearly distinguishes duplications from other gain events, we opted to further filter out any potential duplication events that may have been misclassified as gains by Orthogroups. We used BLAST [47] with default settings to compare all against all *S*. *cerevisiae* proteins. We then considered as potential duplication events cases where a gained protein bares significant sequence similarity (e<10^−4^) to an older gene. Out of 880 genes initially identified as gain events, 84 showed evidence of duplication and were thus discarded from the analysis.

### Controlling for lineage-independent propensity for gene loss

We defined the overall propensity for gene loss as the number of independent loss events divided by the total branch length where a loss could have occurred (see “Estimating relative branch lengths”).

### Estimating relative branch lengths

In order to estimate relative branch lengths along the tree, we selected 3 slowly evolving, universally conserved proteins (UBA1, URA2 and EFT2), calculated the rate of missense substitutions (K_a_) between all pairs of species with PAML 4 [48] and used the median K_a_ as the distance between two species. Then, we calculated the branch lengths in a stepwise manner, starting from the closest pairs of organisms/phyla and progressing upwards along the tree, until the all branch lengths were inferred.

## Supporting Information

S1 Table This table lists *S. cerevisiae* ORFs, their evolutionary classifications, and their various functional and network properties. For ORFs successfully mapped to an *S*. *pombe* ortholog, the name of the ortholog and its properties in *S*. *pombe* are also listed.
(TSV)
